# ‘It is like learning how to live all over again’ A systematic review of people’s experiences of living with a chronic illness from a self-determination theory perspective

**DOI:** 10.1080/21642850.2020.1794879

**Published:** 2020-07-23

**Authors:** Daniela Eassey, Helen K. Reddel, Kath Ryan, Lorraine Smith

**Affiliations:** a School of Pharmacy, Faculty of Medicine and Health, University of Sydney, Sydney, Australia; b Woolcock Institute of Medical Research, University of Sydney, Sydney, Australia; c School of Pharmacy, University of Reading, Reading, UK

**Keywords:** Self-determination theory, autonomy, competence, relatedness, chronic illness, qualitative studies

## Abstract

**Objective:** To conduct a systematic review and synthesis of qualitative evidence exploring the views and experiences of people living with a chronic illness that utilised self-determination theory.

**Methods:** We searched MEDLINE via OvidSP, PsycINFO via OvidSP, PubMed, CINAHL, EMBASE, Google Scholar, the journals Qualitative Health Research and Qualitative Research. Studies were included if they used qualitative methods, explored the subjective experiences of people living with a chronic illness and underpinned the analysis with self-determination theory.

**Results:** From 4605 articles, six met the inclusion criteria. The synthesis culminated in a line of argument that patients endeavoured to be ‘free from disease’. People’s desire to live a life free from disease required nurturing their: (1) need to feel empowered (autonomy), (2) perceived ability to self-care (competence) and, (3) their need to feel a sense of belonging (relatedness).

**Conclusion:** This review has highlighted that the majority of studies included in this review focused on the clinical aspects of managing a chronic condition and changing patient health behaviours. This suggests that there is a need for deep reflection on the current practice of caring for patients with a chronic illness. Exploring the lived experience has the potential to unravel the psychological and emotional needs of those living with a chronic illness.

## Introduction

The incidence of chronic illness, such as diabetes, respiratory and cardiovascular disease, is rapidly increasing and they are among the most prevalent and costly of all global health problems (World Health Organisation (WHO), 2014). According to the WHO, chronic diseases are expected to account for almost three-quarters of all deaths worldwide (WHO, 2019). A chronic illness is defined as a long-term and persistent health condition which may lead to other health complications and may be associated with functional impairment or disability (The Australian Institute for Health and Welfare, 2019).

Given the global burden of chronic illnesses, they have become a priority for healthcare systems around the world. There are strong arguments, however, that current strategies are largely developed to deal with acute episodic care rather than providing care for people with long-term conditions (Reynolds et al., 2018; WHO, 2002). In 2002, the WHO released a report entitled ‘Innovation Care for Chronic Conditions: Building Blocks for action’ (WHO, 2002) and reported the urgent need for action to care for people with chronic conditions (WHO, 2005). Since then, there has been a significant effort to direct national and international policies towards improving the prevention, treatment and ongoing management of chronic illnesses (WHO, 2014).

The WHO, other health policy organisations, and various national governments advocate and endorse the need for health care institutions to place a greater emphasis on the individual (Australian Commission on Safety and Quality in Health Care (ACSQHC), 2011; Delaney, 2018; National Health Service, 2005; US Department of Health and Human Services, 2008). As part of these policy changes, healthcare providers are encouraged to shift from the traditional paternalistic approach of medicine, where medical practitioners prescribe treatment and give instructions with limited input from patients and families, to taking a more patient-centred approach (i.e. more collaborative, responsive to patient’s needs and values and empowering patients’ autonomy) (Bodenheimer, Wagner, & Grumbach, 2002).

To date, the patient-centred model is perceived as part of the solution to paternalism in clinical management (Pulvirenti, McMillan, & Lawn, 2014). This approach is argued to provide increased understanding, confidence in decision-making and improved health-related behaviours (Delaney, 2018). Thus, it is unsurprising that a patient-centred model forms a fundamental principle of self-management of chronic illnesses (Pulvirenti et al., 2014). Self-management integrates numerous concepts and frameworks in research and policy to support health providers as their roles evolve in caring for people living with a chronic illness (Nolte & McKee, 2008).

The concept of supporting ‘patient self-management’ continues to attract significant interest from health policy leaders internationally. Research has shown, however, that there is currently a disconnect between what is recommended in guidelines, what happens in practice and what people living with chronic illness face on a daily basis. Currently, health professionals ‘support’ self-management in a clinical manner by focusing on setting medical regimens (Higgins, Murphy, Worcester, & Daffey, 2012), changing self-care health behaviours and setting medical goals but as a result they may fail to understand patients at an individual level. Research has shown that there is need to provide care more inclusive of psychological and social factors (Entwistle, Cribb, & Owens, 2018). One explanation for this disconnect, could be a lack of research evidence and focus on the patient's experience of living with a chronic illness.

Clearly, a person’s perceptions and experiences of living with a chronic illness are extremely important. Research has shown that personal, environmental and social factors, goals and values all play a role in the ways in which people with chronic illnesses live with their condition (Entwistle et al., 2018). Adopting a more individualised approach to healthcare services would require an exploration of the viewpoints of those concerned and would further provide insight into the needs and values of those affected. This is consistent with the underpinnings of the biopsychosocial model. In this model, the biological, psychological and social processes are integrated and inseparable. The well-being of patients is understood to be influenced by each of these processes (Turabian, 2018). From a biopsychosocial perspective, current approaches to supporting people to live with and manage a chronic illness are understood within the clinical context. This is problematic as it overlooks the broader set of emotional, psychological and social issues people face when living with a chronic illness.

A growing body of theoretical and empirical research emphasises that the effectiveness of biomedical strategies depends significantly on whether and how well they address issues on an individual level by examining the psychological, social and environmental factors that may explain health-related behaviours (Fisher et al., 2005; Institute of Medicine and Board on Health Sciences Policy, 2003; Skovlund & Peyrot, 2005). Self-determination theory (SDT) was developed in the mid-1980s by Professors Richard M. Ryan and Edward L. Deci (Ryan & Deci, 2002; Ryan & Deci, 2017). This theory takes a biopsychosocial approach to explain health related behaviours and motivations on an individual level (Ryan & Deci, 2002; Ryan & Deci, 2017; Williams, Frankel, Campbell, & Deci, 2000). It differentiates between an individual’s intrinsic and extrinsic motivations to initiate changes and does so by incorporating socio-contextual factors which may influence self-motivation (Ryan & Deci, 2002).

Over the past 15 years a growing body of evidence has confirmed the efficacy of this theoretical model in health behaviour change interventions for physical activity, glycaemic control (type 2 diabetes) and medication adherence (DeWalt et al., 2009; Estabrooks et al., 2005; Juul, Maindal, Zoffmann, Frydenberg, & Sandbaek, 2014; Karlsen, Rasmussen Bruun, & Oftedal, 2018; King et al., 2010; Koponen, Simonsen, & Suominen, 2018; Williams et al., 2009). According to this theory, people have three basic psychological needs: autonomy, competence and relatedness. The need for autonomy refers to the experience of behaviour as volitional and self-endorsed. Competence, in turn, refers to feeling a sense of mastery and efficacy in one’s activities. Relatedness reflects the extent to which a person feels accepted, understood and cared for and connected to others (Ryan & Deci, 2017). These psychological needs are perceived as building blocks to intrinsic motivation. Fulfilment of these three needs has been shown to contribute to enhanced motivation and improved health outcomes (Ryan & Deci, 2017). One systematic review quantitatively synthesised the copious empirical studies that have utilised SDT measures in the health care and health promotion contexts (Ng et al., 2012). There is, however, currently no comprehensive systematic review (qualitative or quantitative) that draws on the basic psychological needs identified by SDT in order to examine the personal experiences of people living with a chronic illness, that is, what matters to people and how they live with their chronic illness.

Integrating qualitative research studies into a synthesis will generate new insights into the existing theoretical and empirical work in this area. The objective of this systematic review is to identify, describe and synthesise the views and experiences of people living with a chronic illness from the available qualitative literature that utilised SDT.

## Methods

The review is reported in accordance with the Preferred Reporting Items for Systematic Reviews and Meta-analyses (PRISMA).

### Search strategy

A systematic literature search was conducted in May 2019 in the electronic databases MEDLINE via OvidSP, CINAHL, PsycINFO via OvidSP, PubMed, Scopus and EMBASE from their inception. We also searched Google Scholar, the journals *Qualitative Health Research* and *Qualitative Research* and screened reference lists of relevant studies.

### Selection criteria

Studies were included if they: (1) described qualitative research; (2) explored the subjective experiences of people about living with or managing a chronic illness; and 3)( underpinned their analysis with the three basic psychological needs postulated by SDT. Articles were excluded if: (1) they did not address the personal experiences of living with a chronic illness and (2) due to a lack of resources for translation, were not published in English. In May 2019, a specialist librarian helped design a search strategy with assistance from one of the reviewers (DE). This was then discussed and confirmed with a second reviewer (LS).

### Data extraction

Two reviewers independently (DE, LS) screened titles and abstracts of all identified references. Search results from the different databases were combined and duplicates were removed electronically and manually. Potentially relevant studies underwent full text analysis by two reviewers (DE, LS), differences in opinions were discussed and a consensus was reached. The search strategy is provided in Appendix A.

### Data analysis

Qualitative research is interpretative; therefore, the experiences reported by participants and the original authors’ analytic interpretation of the qualitative findings were extracted verbatim and became the data for the synthesis (Gallacher, May, Montori, & Mair, 2011). We used a framework synthesis which consisted of a two-stage approach (Carroll, Booth, Leaviss, & Rick, 2013; Dixon-Woods, 2011). The first stage of the process required data extraction and organisation into pre-specified categories. These categories were informed by the SDT constructs of autonomy, competence and relatedness. These were then thematically analysed to identify patterns of data within and between categories (Carroll et al., 2013; Miles & Huberman, 1994). A systematic and iterative process was used to derive the final synthesis of the overarching theme. The synthesis was led by DE and LS.

### Reflexivity

Reflexivity is critical to enhancing the quality and credibility of qualitative research, by continuous assessment of the impact of the researcher’s own position and interest on all stages of the research process (Fontana, 2004; Jootun, McGhee, & Marland, 2009). The strategies used to counter potential biases during data extraction and synthesis included independent checking of the original data extraction and synthesis (from DE) by two other experienced reviewers (LS and HR). The process of coding, analysing and interpreting the data involved all authors, who each had experience in qualitative research (DE, LS, KR, HKR).

### Quality assessment

Despite a lack of consensus about quality assessment of qualitative studies, several tools and techniques are available (Leung, 2015). This review used the Critical Appraisal Skills Programme (CASP) tool (CASP, 2018). CASP includes ten questions assessing the rigour, credibility and relevance of the studies (CASP, 2018). This assessment was carried out by one reviewer (DE) and cross checked by a second reviewer (LS) (Table 1).

**Table 1.** CASP checklist results for included studies.

Included Studies	Bingham et al. (2017)	Bingham and Meyer (2011)	Bhattacharya (2012)	Murphy et al. (2015)	Knudsen et al. (2018)	Eassey et al. (2019)
CASP questions						
Q1-Was there a clear statement of the aims of the research?	No	Yes	Yes	Yes	Yes	Yes
Q2-Is a qualitative methodology appropriate	Yes	Yes	Yes	Yes	Yes	Yes
Q3-Was the research design appropriate to address the aims of the research?	Yes	Yes	Yes	Yes	Yes	Yes
Q4-Was the recruitment strategy appropriate to the aims of the research?	Yes	Yes	Yes	Yes	Yes	Yes
Q5- Was the data collected in a way that addressed the research issue?	Yes	Yes	Yes	Yes	Yes	Yes
Q6- Has the relationship between researcher and participants been adequately considered?	No	No	No	Yes	Yes	Yes
Q7- Have ethical issues been taken into consideration?	No	No	Yes	Yes	Yes	Yes
Q8- Was the data analysis sufficiently rigorous?	Yes	Yes	Yes	Yes	Yes	Yes
Q9-Is there a clear statement of findings?	Yes	Yes	Yes	Yes	Yes	Yes
Q10- How valuable is the research?	Yes	Yes	Yes	Yes	Yes	Yes

## Results

### Description of the studies

**Figure 1.**
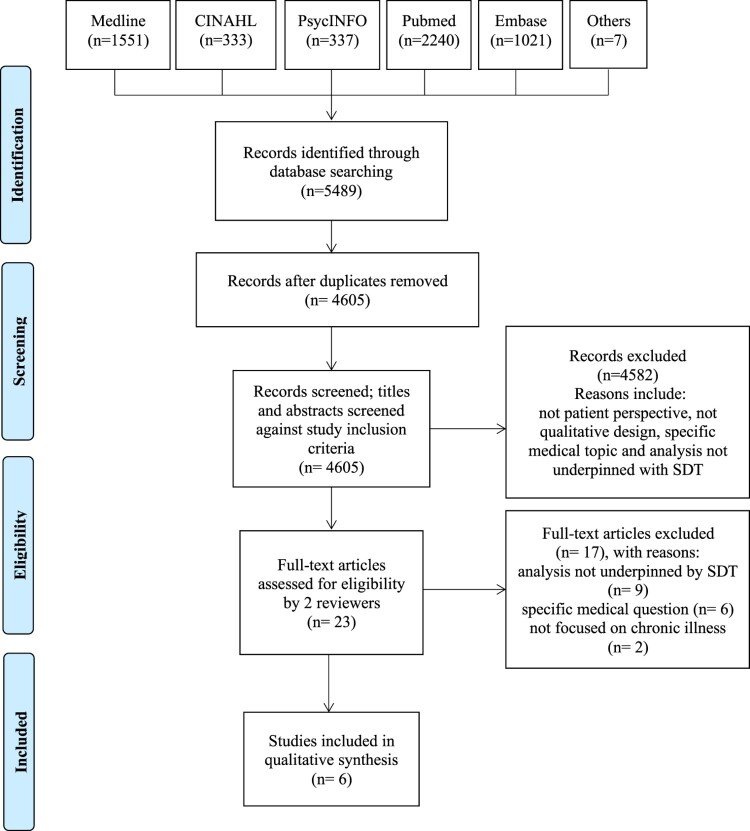
Flow of information through the different phases of the systematic review (based on the PRISMA reporting flowchart).

From the 4605 potentially relevant studies, twenty-three investigated people’s experiences of living with a chronic illness, used qualitative methods, and mentioned SDT in the abstract. After full text review, six articles were included, that had based their analyses on the principles of SDT. Figure 1 provides a PRISMA flow diagram indicating the inclusion and exclusion of articles at each stage of the screening process. The included papers were published between 2011 and 2019, from Australia (Eassey, Reddel, Ryan, & Smith, 2019), the Netherlands (Knudsen et al., 2018), South Africa (Murphy, Chuma, Mathews, Steyn, & Levitt, 2015) and the United States (Bhattacharya, 2012; Bingham & Meyer, 2011; Bingham, Crane, & Manning, 2017). The included papers described people’s experiences of living with: cystic fibrosis (Bingham & Meyer, 2011; Knudsen et al., 2018), asthma (Bingham et al., 2017; Eassey et al., 2019), diabetes (Bhattacharya, 2012) and hypertension (Murphy et al., 2015).

The majority of studies did not focus on what matters to people in their everyday experiences of living with a chronic illness but rather more specifically on barriers to self-management (Bhattacharya, 2012; Bingham et al., 2017; Bingham & Meyer, 2011; Knudsen et al., 2018; Murphy et al., 2015). Of the six studies, four aimed to identify factors that could be used in subsequent intervention studies by focusing on the challenges to patients’ basic psychological needs (Bhattacharya, 2012; Bingham et al., 2017; Bingham & Meyer, 2011; Murphy et al., 2015), one study explored patient experiences of participating in a life coaching intervention (Knudsen et al., 2018) and one explored in depth the role of autonomy in experiences of living with a debilitating condition (Eassey et al., 2019). Five studies reported on all three of SDT’s basic psychological needs (autonomy, competence and relatedness) (Bhattacharya, 2012; Bingham et al., 2017; Bingham & Meyer, 2011; Knudsen et al., 2018; Murphy et al., 2015) and one study analysed and interpreted one of the needs (autonomy) in depth using people’s narratives (Eassey et al., 2019). A range of qualitative analysis methods was used; all sought to underpin their analysis or interpretation with SDT’s theory of basic psychological needs. Details of the included studies are presented in Table 2.

**Table 2.** Summary of included studies.

Authors	Country	Patients (n)	Sex (M/F)	Age Range (y)	Patient population	Data collection	Data analysis	Self-determination theoretical framework	Research topics and scope
Children and adolescents									
Bingham & Meyer (2011)	USA	17	10/7	8–16	Children living with Cystic Fibrosis (CF)	Structured interviews and SDT survey	Categorisation	BPN part of analytical approach	1) Barriers to adherence, 2) Attitudes about CF, 3) Healthcare experience Influences of CF self-management
Bingham et al. (2017)	USA	15	13/2	7–17	Children living with asthma	Semi-structured interviews	Categorisation and deductive analysis from interview questions	Intrinsic and Extrinsic motivation and BPN part of the analytical approach and used to interpret findings	1) Illness experience, 2) Preferred recreational activities (non-digital and digital), 3) Motivation to improve asthma symptoms
Adults									
Bhattacharya (2012)	USA	31	15/16	Age group % reported: 25 to >65 years	Rural African Americans living with Type 2 Diabetes (T2D)	Narrative approach Semi-structured interview	Thematic analysis (selective coding)	BPN used to interpret findings	1) Intrinsic and extrinsic motivation, 2) Influences upon T2D self-management
Murphy et al. (2015)	Africa (South)	22	6/16	35–75	People living with diabetes and hypertension	In-depth interviews	Inductive and Deductive analysis Categorisation	BPN part of analytical approach	1) Patient perceived motivation 2) capacity for self-management and lifestyle change
Knudsen et al. (2018)	Netherlands	14	4/10	18–30	Young adults living with Cystic Fibrosis	Semi-structured interviews	Thematic analysis	BPN used to interpret findings but not part of analytic approach	Experiences of life coaching intervention for those living with Cystic Fibrosis** Illness experience
Eassey et al. (2019)	Australia	29	14/15	19–74	People living with severe asthma	Narrative approach Semi-structured interviews Follow-up open-ended questions	Inductive and deductive analysis Thematic analysis	BPN- in depth exploration on the role of autonomy	1) Illness experience, 2) Role of autonomy

NS = Not Stated.

BPN = Basic Psychological Needs.

OIT = Organismic Integration Theory.

** Although this study was exploring the experiences of life coaching for patients living with CF there were sections in the analysis which noted their illness experience. Thus, this review included the sections on people’s views and experiences of living with CF and excluded sections on perceptions of the life coaching.

### Synthesis of findings

**Figure 2.**
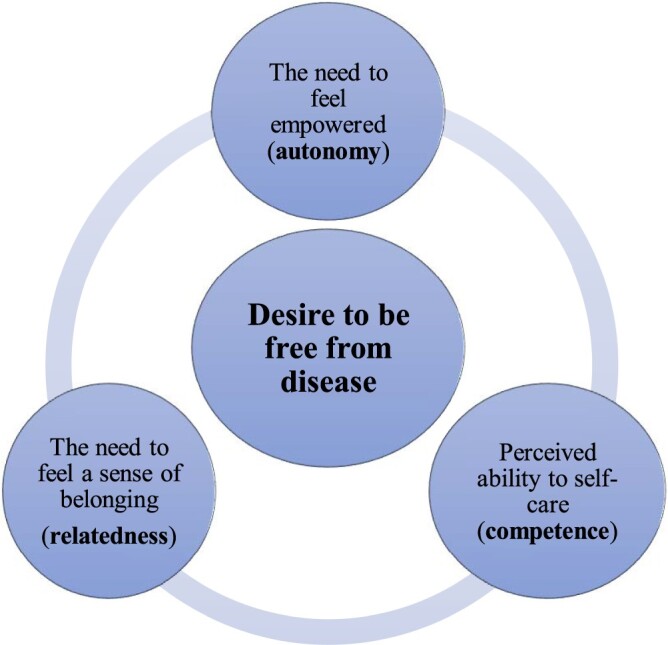
Conceptual diagram of the overarching theme and the sub-categories using framework synthesis.

Our synthesis of the included studies culminated in a line of argument that patients endeavoured to be ‘free from disease’. A chronic illness was considered by patients to be a ‘disease’ if it challenged their sense of autonomy, competence and/or relatedness. It was apparent that in their attempt and desire to *live a life free from disease*, people were seeking ways to understand their condition and learn ‘how to live all over again’ (Bhattacharya, 2012). People’s desire to live a life free from disease required nurturing their: (1) need to feel empowered (autonomy), (2) perceived ability to self-care (competence) and, (3) their need to feel a sense of belonging (relatedness) (Figure 2).

### The need to feel empowered (autonomy)

This subtheme describes that, in their desire to live a ‘life free from disease’, people valued feeling ‘empowered'. The conflict between the constraints of their illness and the desire to live a life free from disease was a constant ‘power’ battle. Feeling empowered meant having a sense of personal control over their condition and a sense of choice in health-related decisions. The authors of the included studies described a lack of personal control and lack of choice over health decisions and their condition as a ‘barrier to autonomy’ (Bhattacharya, 2012; Knudsen et al., 2018; Murphy et al., 2015). One author further suggested that ‘the lack of autonomy meant negative [health] outcomes were inevitable to participants’ (Bhattacharya, 2012).

### Feeling empowered over making health-related decisions

People reported the extent to which they felt their healthcare providers provided them with a sense of personal control and choice over their health-related decisions (Eassey et al., 2019; Murphy et al., 2015). Murphy et al., (Murphy et al., 2015) highlighted that none of their participants felt that their healthcare providers actively encouraged their collaboration. It appeared that people felt that they were not provided with enough information to engage in decisions related to their health. For example, one participant reported ‘I am not sure what it (diabetes) is, but at least what I am doing is just to always get my medications’ (Murphy et al., 2015).

The traditional biomedical model of providing information without any interaction with the patient, made people feel apprehensive about asking questions:

I am not happy. I feel dizzy and tired … . I think that this is the wrong medication. I wish that the doctor can change that tablet that I am using … Even today, I was telling myself to ask the doctor if she can change my medication, but I am worried about what she will say to me … (Murphy et al., 2015)

People also reported actively opposing the perceived controlling efforts of their healthcare providers, even if this could have negative health implications: ‘I was told that I was not to have budgies because they carry bird fancier’s disease. Yeah but his cage gets kept clean and he runs the house. So, what my specialist doesn’t know won’t hurt me.’ (Eassey et al., 2019).

People reported that they valued making autonomous decisions which at times conflicted with recommended guidelines for optimising health (Knudsen et al., 2018). All but one study reported that, as a result of the lack of choice and collaboration, some participants exerted their personal control by deciding not to take their medications strictly as directed, or as the authors described it as ‘being non-adherent’ (Bhattacharya, 2012; Bingham et al., 2017; Bingham & Meyer, 2011; Murphy et al., 2015). Pushing back against medical paternalism was perceived by patients as enacting their autonomy. According to Bhattacharya, non-adherence was interpreted as ‘feelings of helplessness [that] could have led to perceived failure of positive behaviour changes outcome’ (Bhattacharya, 2012). Authors described various explanations for non-adherence, such as cultural beliefs where ‘[participants] divulged that health beliefs in African American social cultural contexts connect using medicine with [living with an] illness’ (Bhattacharya, 2012) and perceived impact of taking medications on daily life where, for example, taking medications seemed time consuming (Bingham et al., 2017; Bingham & Meyer, 2011; Eassey et al., 2019; Knudsen et al., 2018).

Studies which reported on the experiences of children living with a chronic illness, suggested that choice was given to the parents; thus, to carry out their treatment routines required a ‘sense of imposed necessity’, for example ‘parents’ insistence, bribes and punishments’ (Bingham et al., 2017; Bingham & Meyer, 2011). Adolescents with cystic fibrosis (for which the daily treatment is very time-consuming) seemed to prioritise life choices over their illness, in that they chose going out with friends or going to work over taking their medications, disclosing that not taking their medication was often intentional: ‘I never forget to take my medication, I opt out [of] my medication. If I don’t take it, [that’s] because I have consciously chosen not to take it today … ’ (Knudsen et al., 2018). Another adolescent reported:

Bad conscience is nagging me, there is always more you could do. But I have made a choice, I don’t know if it is the right one, but I have chosen that (the disease) should not restrict me, meaning that if I must choose between taking my treatment or doing something with my friends, then I will without doubt choose my friends. (Knudsen et al., 2018)

Adults expressed a range of feelings about adherence to treatments. They felt worried having to take medications and to monitor their condition for the rest of their lives, some suggesting that the illness had become a *disease* in that it had taken control over their lives. One study, however, described that health-related behaviours such as taking medication were valued as a way to maintain a sense of control over their condition (Eassey et al., 2019). For example, one person reported that he saw not having his medications as a potential threat to his sense of personal control: ‘I’m out of control, I start to worry. But if you’ve got everything there you need to control it, you don’t worry … nothing worse than not being in control of your body, not being able to breathe … ’ (Eassey et al., 2019).

It was also noted that, in people’s desire to live a life free from disease, negative emotions such as fear and helplessness played a role in their sense of control over their condition. The authors of two studies had opposing views on the influence of emotions on engagement in health-related behaviours such taking medication or making lifestyle changes (Bhattacharya, 2012; Eassey et al., 2019). On the one hand, one study reported that people’s sense of helplessness led to the failure of positive health behaviour changes such as taking medications regularly (Bhattacharya, 2012), whilst on the other hand another study reported that negative emotions such as fear, despite challenging people’s sense of personal control, were a ‘strong motivator to engage in health-related behaviours and exert personal control.’ (Eassey et al., 2019).

### Feeling able to resist the controlling effect of their condition

One study looked at the role of autonomy in depth through the narratives of people living with severe asthma. The authors suggested that patients’ autonomy was either challenged or enacted in a range of situations such as: ‘dealing with threats to self-identity and in living an ‘unconstrained’ life’ (Eassey et al., 2019). People described that maintaining employment whilst living with their condition was a highly valued aspect of life. In their desire to live a life free from disease, many examples emerged of people pushing their own personal limits to resist perceived controlling effects of their condition. For example, one participant reported that ‘there were days he should have stayed home due to his condition, but he decided to push through and go to work’ (Eassey et al., 2019).

People also reported that when they felt their identity confronted by the illness, they would ‘push back’ to limit the effects of the illness (Eassey et al., 2019). One person reported that due to being in and out of hospital, she felt that she could not fulfil her role of being a mother:

You know, you’ve got kids that want to play with you, and you can’t and the worst thing has been the amount of times I have been in hospital when it’s my kids’ birthdays or I’ve just been too sick … we try and make up for it, you know, I buy way too much stuff to try and make up for it … Thank you credit card … but yeah, you live in fear all the time, of so many things, and you live hating how you’re failing your family. I want to be able to take my kids out. I’ve always wanted to be able to. (Eassey et al., 2019)

According to the authors, autonomy was also enacted when trying to search for a ‘new normality’ or preserving their sense of normality (Eassey et al., 2019). For some, this required constant negotiation and reconceptualisation by incorporating the illness into their lives.

## Perceived ability to self-care (competence)

The authors of the included studies described competence as an individual’s sense of confidence in their ability to engage in behaviour changes (self-care) (Bhattacharya, 2012; Bingham et al., 2017; Bingham & Meyer, 2011; Knudsen et al., 2018; Murphy et al., 2015). They focused on clinical issues hindering self-care practices. According to the authors, self-care meant understanding the participant’s knowledge about their condition and barriers to making lifestyle changes (Bhattacharya, 2012; Bingham et al., 2017; Bingham & Meyer, 2011; Knudsen et al., 2018; Murphy et al., 2015).

### People’s need to feel effective in understanding their body

People reported on the importance of understanding their condition through being aware of symptoms and the power of knowledge about their illness. For children living with asthma, being aware symptoms such as ‘wheeziness, tightness and cough’(Bingham et al., 2017) made them feel able to identify their condition. The authors suggested that being provided with information and knowledge by their healthcare providers was a core component for patients to feel competent to self-manage (Bingham et al., 2017; Murphy et al., 2015).

A study by Murphy et al., (Murphy et al., 2015) highlighted that many people with diabetes or hypertension had low health literacy. They suggested that there was a lack of information provided by healthcare providers, and that ‘*without* an attempt on their part to understand patients’ existing levels of knowledge or beliefs about their illness, problematic misconceptions [by patients] about the causes of Type 2 Diabetes and hypertension prevailed’. For example, a participant reported that their hypertension was inherited and that they could have done nothing to avoid it: ‘My grandmother used to have high blood pressure. So, the day that I was diagnosed, I was waiting for it’ (Murphy et al., 2015). Due to the lack of understanding about their condition, another participant reported using alternative remedies to cure their illness such as ‘holy water’, Aloe Vera or other strategies. For example: ‘There is a lady who told me that this (diabetes) is caused by dirty blood. She told me that I must clean my blood with a Vitamin B injection from the chemist and then my blood will be okay’ (Murphy et al., 2015). Furthermore, for children, Bingham et al.,(Bingham et al., 2017) suggested that linguistic competence may influence their perceived competence to manage their condition. All these factors had ‘negative implications for self-care’ (Murphy et al., 2015).

### The need to feel effective in changing lifestyle behaviours

In their desire to live a life free from disease, participants felt conflicted with the need to feel free versus having to follow strict regimens such as changing lifestyle behaviours. The authors of the included papers described the need for competence as the ability to understand and follow the pharmacological and non-pharmacological advice given by their doctors (Bhattacharya, 2012; Bingham et al., 2017; Bingham & Meyer, 2011; Knudsen et al., 2018; Murphy et al., 2015). Lifestyle changes such as: ‘exercising, sleeping well, eating health[ily] and avoiding too much alcohol’ (Knudsen et al., 2018), were described by the authors as the ‘individual’s responsibility’ (Bhattacharya, 2012). There was an emotional disconnect between how people perceived ‘managing’ their condition and the recommended lifestyle changes. For example, one author suggested that this disconnect was due to some participants feeling ‘disrespected’ by healthcare providers; where they felt that their healthcare providers focused on guidelines which deemed not practical in their everyday life (Bhattacharya, 2012).

According to Bhattacharya, participants ‘lacked self-competence to follow guidelines’ (Bhattacharya, 2012). Following instructions given by healthcare providers, such as changing diet, at times was perceived as challenging due to people’s cultural upbringing in which food and dietary patterns ‘had been practiced from their grandmother’s time’ (Bhattacharya, 2012). Other authors suggested that patients were willing to make lifestyle changes but needed more information from their healthcare providers. As one patient reported:

The doctor said that I must leave (out) the salt in the food because the danger is the salt. But she didn’t explain what kind of food I must eat. So, I don’t know what I must eat at home … (Murphy et al., 2015).

People also reported feeling more confident in their ability to make decisions and cope with their condition when they searched for more information on lifestyle changes and medication from other sources: ‘From the day hospital, I went onto the internet, read books, um, talked to other people. I found out a lot of the things … ’ (Murphy et al., 2015). However, being told what to do by their healthcare providers, with no context about the reality of their lives, contributed to people feeling helpless (Bhattacharya, 2012). The authors described that feeling helpless ‘hampered [people’s] self-motivation to make [health related behavioural] changes’ (Bhattacharya, 2012).

## The need to feel a sense of belonging (relatedness)

People reported wanting to live a ‘normal life’, or life that they felt was not controlled by their disease. Many examples indicated that to live a ‘normal life’, participants felt it was important to develop a sense of connection (or relatedness) with others. The authors mainly focused on barriers to people’s sense of relatedness and its impact on their health behaviours (Bhattacharya, 2012; Bingham et al., 2017; Bingham & Meyer, 2011; Knudsen et al., 2018; Murphy et al., 2015). Across the studies, relatedness was defined as ‘feelings of social support and connectedness to boost [people’s] intrinsic beliefs in [their] capacity to make health related behaviour changes’ (Bhattacharya, 2012). Living a normal life meant not feeling judged or different, being cared for, and understood by their healthcare providers, social contacts and immediate family.

### Interactions with healthcare providers

Interactions with healthcare providers were important for people to feel that they were living a ‘normal life’ rather than living a life with a disease. The authors of one study reported on the barriers that affected ‘patients’ ability to accept their condition, as well as their motivation to engage with healthcare providers’ (Murphy et al., 2015). One study reported on people’s sense of relatedness with their healthcare providers (Murphy et al., 2015). According to Murphy et al. (2015) participants reported wanting to feel understood, cared for and valued by their healthcare providers.

The authors noted that participants’ experiences pointed to ‘a number of issues which suggested that providers had failed to establish a sense of relatedness with them’ (Murphy et al., 2015). Participants also reported that healthcare providers did not have enough time with them. One participant reported: ‘I know the doctors are under pressure and there’s the next patient waiting, but I just wish that they could be more detailed, sort of give us more information, talk with us about what this whole thing entails.' (Murphy et al., 2015). Other reasons provided by authors were

healthcare providers failing to explain the cause and nature of their illness and counsel [patients] on self-management, a lack of emotional support and reassurance for patients, particularly at the time of diagnosis and accounts of providers alienating patients by being impatient and rude. (Murphy et al., 2015)

Participants also described how non-empathetic attitudes of some healthcare providers inhibited them from being open and trusting. For example, one participant reported:

when it comes to the nurses it is really bad. When you go inside to see the doctor, he will talk to you fine, but when it comes to the nurses, hm-mm, they are so rude. It’s not a good experience. (Murphy et al., 2015).

### Interactions with their social contacts

All but one of the included studies reported that people living with a chronic illness were concerned about feeling left out by their peers (Bhattacharya, 2012; Bingham et al., 2017; Bingham & Meyer, 2011; Knudsen et al., 2018; Murphy et al., 2015). Interviewees stated that social isolation thwarted their sense of ‘normality’ (Knudsen et al., 2018). This may be due to the physical challenges they faced with their condition or the requirements of their treatment or the ‘stigma’ related to their condition.

Children with asthma reported a range of reasons for feeling left out by their friends such as having to miss school days or feeling tyred due to the lack of sleep (Bingham et al., 2017). Having friends with the same condition was reported to be a ‘ positive influence’ because they felt able to relate to one another and were able to ‘take a break [such as missing events] without feeling left out’ (Bingham et al., 2017). Other reasons identified were associated with time-consuming treatment regimens. For example, as one author reported, people living with cystic fibrosis used ‘masks for delivering nebulised medicines which stopped [them] from talking and [a] vibratory vest kept them from hearing.' Yet, despite these barriers ‘participants considered themselves similar to their peers.' (Bingham & Meyer, 2011)

A few children, commented on how they didn’t want to be perceived as ‘weak' or ‘an outcast' (Bingham et al., 2017). Participants wanted to develop close and supportive relationships with other people yet were cautious about disclosing their illness to others (Knudsen et al., 2018). Participants reported being cautious, due to the fear of ‘stigmatisation' (Bhattacharya, 2012; Knudsen et al., 2018). One participant said ‘‘It is a rural community, word will spread, and all will look at me with sympathy’’(Bhattacharya, 2012). When people with diabetes were asked whether they were aware of anyone else within their [rural] community living with the same condition, they responded that they learned it ‘through the grapevine’ (Bhattacharya, 2012). Gender also played a role in the responsibility of having to take care of their family. Interviewees considered how going on a ‘special diet’, taking medication and having to exercise would be perceived by their family members. ‘How can I say I am now sick for my whole life? They will think who will take care of this man?’ (Bhattacharya, 2012). Another participant reported: ‘My family is young – young kids, wife. They may not say much but think I am a sick person.’ (Bhattacharya, 2012). According to the authors, people’s perceptions of how they were viewed by their social environment ‘affected their ability to carry out lifestyle changes’(Bhattacharya, 2012).

One author highlighted the cultural challenges for African-Americans living with a chronic illness (diabetes) in a rural area (Bhattacharya, 2012). They reported on the spiritual aspects of going to church and its impact on coping with their chronic illness. Praying offered some people a moment of relief and assurance. One person reported feeling as if they were living free from disease and that ‘god [would] take care of them’. However, another participant reported:

I do not believe church can do anything for my diabetes care. Church is the sacred place to worship God. I come to the church to pray to God. I do not want to bring up my problems, worries, and anxieties in the church. I want peace and quietness in the church to be with God-spiritually. (Bhattacharya, 2012)

Feeling a connection with a higher order provided patients with a sense of relatedness, where they felt cared for and understood at a spiritual level. For example, one person reported that they were at the mercy of God:

I worry a lot about my diabetes and losing [my] legs, and maybe earlier death. Doctor told me to prick myself three times a day to test blood sugar. But I believe in destiny. I left it to God. When He thinks it is my time to leave this world, I will be ready to go! (Bhattacharya, 2012)

## Discussion

To our knowledge, this is the first systematic review to examine qualitative studies drawing on self-determination theory (SDT) as a framework to understand people’s experiences of living with and managing a chronic condition. The key theme emerging from our analysis of the six identified studies is that people living with chronic illness want *to live a life free from disease*. Our synthesis captured that, in their desire to live a life free from disease, people faced constant psychological and emotional challenges to their sense of autonomy, competence and relatedness.

At the heart of concepts such as self-management and patient-centred care is autonomy. According to SDT, autonomy is a concept central to satisfying basic psychological needs for wellbeing (Ryan & Deci, 2002; Ryan & Deci, 2017). Our findings go beyond those of previous quantitative studies that demonstrated how supporting patient autonomy improves health outcomes (Chirkov, Ryan, Kim, & Kaplan, 2003; Deci & Ryan, 2012; Ng et al., 2012; Ryan & Deci, 2000). This review has highlighted that, while the majority of included studies focused on barriers to self-management such as lack of personal choice over health decisions and feeling misunderstood by their healthcare providers; only one study reported how autonomy was challenged and enacted to preserve people’s sense of identity (Eassey et al., 2019). This resonates with the work conducted by Charmaz on the impact of living with a chronic illness on an individual’s sense of identity (Charmaz, 1983; Charmaz, 1995). Charmaz suggested that living with a chronic illness could threaten the unity between the body and the self. She further suggested that learning to adapt or to live well with a condition required resolving the tension between the body and the self, and this required a sense of autonomy (Charmaz, 1983; Charmaz, 1995). We suggest that future studies drawing on the SDT concept of autonomy should consider broader issues such as the psychological effort required for patients to enact their sense of autonomy and preserve their sense of identity.

According to Ryan et al., (Ryan, Patrick, Deci, & Williams, 2008) the effectiveness of most health care interventions depends on people’s perceived competence to undertake self-care activities such as lifestyle changes and symptom management. As an important element of self-management, self-care involves the ability both to care for oneself and to achieve, maintain and promote optimal health (Greaves & Campbell, 2007; Pickard & Rogers, 2012). The authors of the included studies suggested that empowering patients to feel competent in their self-care practices depended on their healthcare professionals providing enough information or knowledge about their condition to allow the patients to make health-related decisions, and on acknowledging people’s cultural background, health literacy and, for children, their linguistic competence. Our findings highlight that people’s decisions to look after their condition, such as lifestyle behaviours, also depend on their relationships with their healthcare providers, friends and family and their overall illness experience. We note, however, that much of the applied research in self-care and self-management has focused on clinical concepts such as improving symptom management and treatment adherence. An integral part of living with a chronic illness is setting goals (DeWalt et al., 2009; Estabrooks et al., 2005; Lenzen, Daniëls, van Bokhoven, van der Weijden, & Beurskens, 2017; Williams, Steven, & Sullivan, 2011). Thus, we note that in light of these findings future studies should consider broader aspects of living with a chronic illness such as examining the role of competence in goal setting, which may enhance our understanding of the psychological effort required to live with an illness.

Social contacts play an important role in shaping people’s illness experience. Most of the studies included in this review focused on the impact of relatedness on self-management. Our synthesis highlights that in their desire to feel ‘normal’, patients valued having a supportive relationship with their healthcare providers, friends and family. Interviewees described that their values and beliefs were not being acknowledged by their healthcare providers. Healthcare providers tended to adopt a paternalistic approach to treating the medical condition rather than considering patients’ psychological needs and overall illness experience. Feeling unable to relate to their healthcare providers or other individuals hindered people’s sense of relatedness. The psychological effort required to feel ‘normal’ resulted in some people feeling helpless and emotionally disconnected. According to Turner and Kelly (2000) the emotional dimensions of living with a chronic condition are frequently overlooked. Participants reported fearing the stigma related to their condition and as a response isolated themselves socially. One study reported that people turned to religion for acceptance and emotional support (Bhattacharya, 2012). According to Pachankis et al. (2017) when people feel stigmatised by others an aspect of the self is socially devalued.

This systematic review adds depth to the current literature drawing on SDT in the health domain. Despite the recent growth of studies encouraging the use of taking an SDT approach in health (Migliorini, Cardinali, & Rania, 2019) or empirically testing the utility of this theory (Martinez et al., 2016; Ng et al., 2012), this review demonstrates the paucity of relevant qualitative research. This is important because qualitative research provides rich information and understanding of the lived experiences of chronic illness. SDT is couched within a biopsychosocial model yet our findings have highlighted that studies examining or implementing this theory have focused on clinical issues such as barriers that might hinder self-management. Our findings illustrate that the included studies emphasise fixing ‘issues’ that are perceived to hinder people from managing their condition. However, we suggest that what matters to patients in terms of their desires to manage (or live well with) their chronic illness has been overlooked (Entwistle et al., 2018; Entwistle & Cribb, 2013; Entwistle, Carter, Cribb, & McCaffery, 2010). Our findings indicate the need to move beyond the narrow approach underpinned by medical paternalism, and pre-determined medical goals, towards taking a broader approach to understanding what it means to live well with a long-term condition.

### Strengths and limitations

This review used a systematic and comprehensive approach using an SDT framework to identify and synthesise qualitative studies examining people’s experiences of living with a chronic illness. There are a few limitations to acknowledge. First, the number of studies identified for this review was small and was limited by the original reporting of data by authors and the selection of quotes used in their papers. We suggest that this reflects the paucity of qualitative research based on SDT in this field, although it is possible that relevant studies may have been missed if SDT was not mentioned in the abstract. Another limitation was that some of the included studies, according to the CASP qualitative checklist, were of only moderate quality (*n* = 3). Further, some were evaluation studies (*n* = 1) or focused on designing an intervention (*n* =  2) suggesting that they were focused on understanding outcomes rather than people’s diverse experiences. This review highlighted that the topics of the included papers were notably clinically focused which is indicative of the type of studies that have been conducted in this area. Our findings suggest that more attention could be paid to the nuances surrounding living with a chronic illness.

## Conclusion

In this first systematic review and synthesis of qualitative literature based on SDT, we propose that people living with a chronic illness desire to live a life free from disease. Our synthesis has highlighted that the majority of studies included in this review focused on *how* and *why* people living with a chronic illness are ‘unable’ to manage their condition and change their health behaviours. Given the theoretical underpinnings of the SDT concept of basic psychological needs, we recommend exploring people’s illness experiences more broadly to develop further understanding of the role of each construct, rather than focusing only on pharmacological interventions.

## Appendix

### Appendix A – search strategy for embase database

Search Terms*

1. Chronic Disease/
2. (chronic and (condition* or illness*)).mp. [mp = title, abstract, heading word, drug trade name, original title, device manufacturer, drug manufacturer, device trade name, keyword, floating subheading word, candidate term word]
3. Diabetes Mellitus/
4. Lung disease/
5. Exp Asthma/
6. Exp Chronic obstructive lung disease/
7. Exp Musculoskeletal disease/
8. Arthritis/
9. Osteoporosis/
10. Degenerative disease/
11. Epilepsy/
12. Mental health /
13. Cardiovascular diseases/
14. Chronic kidney disease.mp./ or exp chronic renal failure/
15. Chronic renal disease.mp.
16. 1 or 2 or 3 or 4 or 5 or 6 or 7 or 8 or 9 or 10 or 11 or 12 or 13 or 14 or 15
17. exp Life Experiences/ or patient experience.mp. or exp Patients/
18. ‘Quality of Life’/
19. ((life or patient* or liv* or personal or health* or illness) adj3 (experience* or perspective* or attitude*)).mp.
20. Attitude/
21. 17 or 18 or 19 or 20
22. (self-determination adj3 theory).mp.
23. Personal Autonomy/
24. (autonom* or competenc* or relatedness).mp. [mp = title, abstract, heading word, drug trade name, original title, device manufacturer, drug manufacturer, device trade name, keyword, floating subheading word, candidate term word]
25. 22 or 23 or 24
26. qualitative.mp. or exp qualitative analysis/ or exp qualitative research
27. (Surveys and Questionnaires).mp. [mp = title, abstract, heading word, drug trade name, original title, device manufacturer, drug manufacturer, device trade name, keyword, floating subheading word, candidate term word]
28. Narrati*.mp. [mp = title, abstract, heading word, drug trade name, original title, device manufacturer, drug manufacturer, de Surveys and Questionnaires vice trade name, keyword, floating subheading word, candidate term word]
29. semi structured interview/ or interview/
30. 26 or 27 or 28 or 29
31. 16 and 21 and 25 and 30
  *this was adapted according to the indexing systems of the other databases searched
